# Characteristic of complete mitochondrial genome and phylogenetic relationship of a Chinese Serow in Xishui, China

**DOI:** 10.1080/23802359.2020.1719934

**Published:** 2020-02-13

**Authors:** Yuhan Wu, Chongqing Li, Yongfang Yao, Huailiang Xu

**Affiliations:** a College of Life Science, Sichuan Agricultural University, Ya’an, Sichuan, P. R. China;; b Xishui National Nature Reserve Administration, Xishui, Guizhou, P. R. China

**Keywords:** Chinese Serow, mitochondrial genome, phylogenetic analysis, Xishui

## Abstract

In this study, we reported a complete mitochondrial genome of Chinese Serow from Guiyang. It has a circular genome of 16,442 bp including 13 protein-coding genes (PCGs), 22 transfer RNA (tRNA) genes, 2 ribosomal RNA genes, and a control region. In the phylogenetic tree, we found it clustered tightly with a sequence with a distinct white forehead and white face outlook in the genus *Capricornis*, which indicated there are a certain number of this species.

There are six species in the *Capricornis* genus Bovidae and it is well recognized that there are only three *Capricornis* species distributed in China including Taiwan Serow, Himalayan Serow, and Chinese Serow (*Capricornis milneedwardsii*) (Smith and Xie 2009). Chinese Serow, a middle herbivore, is distributed in forests with altitude ranging from 1000–4400 m in china and some southeast Asian countries (Hassanin et al. 2009). Taiwan Serow and Himalayan Serow are limited in Taiwan island and Tibet, respectively. Although there are a fair number of Chinese Serows, their population continues to decrease due to hunting and trade, and this species is classified as Near Threatened (NT) on the IUCN Red List (Duckworth et al. 2008), and also on the list of the Class II national protected species in China (Wang and Xie 2008). However, the diversity research of Chinese Serow is limited due to the steep slopes habitat. Therefore, it is urgently needed to obtain more sequences to support conservation of this species.

Herein, we collected fecal samples from China, Guizhou province, Xishui county(N28°32′58′′, E106°.22′16′′). All fieldwork and collection were granted permission by the Administration for Wild Animal and Plant Protection and Nature Reserves and the Department of Forestry for the Guiyang province. Samples were deposited at the Museum of Sichuan Agricultural University (Accession: 000755). Total DNA was extracted using a QIAamp DNA Stool Mini Kit (QIAGEN, Hilden, Germany), following the manufacturer’s instructions. According to the published Chinese Serow mitochondrial (mt) genome sequence (Genebank: FJ207534), we designed 15 pairs of primer and amplified Chinese Serow mt genome sequence successfully.

The newly generated mitochondrial genome (Genebank:MN635784) of Chinese Serow contained 16,442 base pairs, in detail the composition is A = 33.44%, G = 13.20%, T = 26.72%, and C = 26.63%, showing a strong AT preference. The mitochondrion structure of this Chinese Serow includes 13 protein-coding genes (PCGs), 22 transfer RNA (tRNA) genes, 2 ribosomal RNA (rRNA) genes, and a control region (D-loop) in the order typically found in mammals. Among the 13 PCGs, most PCGs (10 of 13) used ATG as start codon while *NAD2* and *NAD3* started with ATA, and *NAD5* started with ATT. Eight PCGs terminated with TAA, TAG or AGA. Especially, when compared with the reference genome we found a three base pair insert in ATPase 8.

To investigate the phylogenetic relationship of Chinese Serow, we conduct a maximum-likelihood (ML) phylogenetic tree using an alignment comprising of 11 sequences from genus *Capricornis* and a *Hemitragus jemlahicus* as a outgroup (Figure 1). The ML tree was generated using MEGA 10 with the 1000 ultrafast bootstrap replicates as default parameters (Kumar et al. 2018). We found our sequence clustered with a sequence from Guiyang, which has been reported with a distinct white forehead and white face outlook in the genus *Capricornis* and is suspected as a new species of *Capricornis* (Dou et al. 2016). Our result indicates there might be a certain number of this species, but we need more investigation to further confirm the taxonomy of this species.

**Figure 1.**
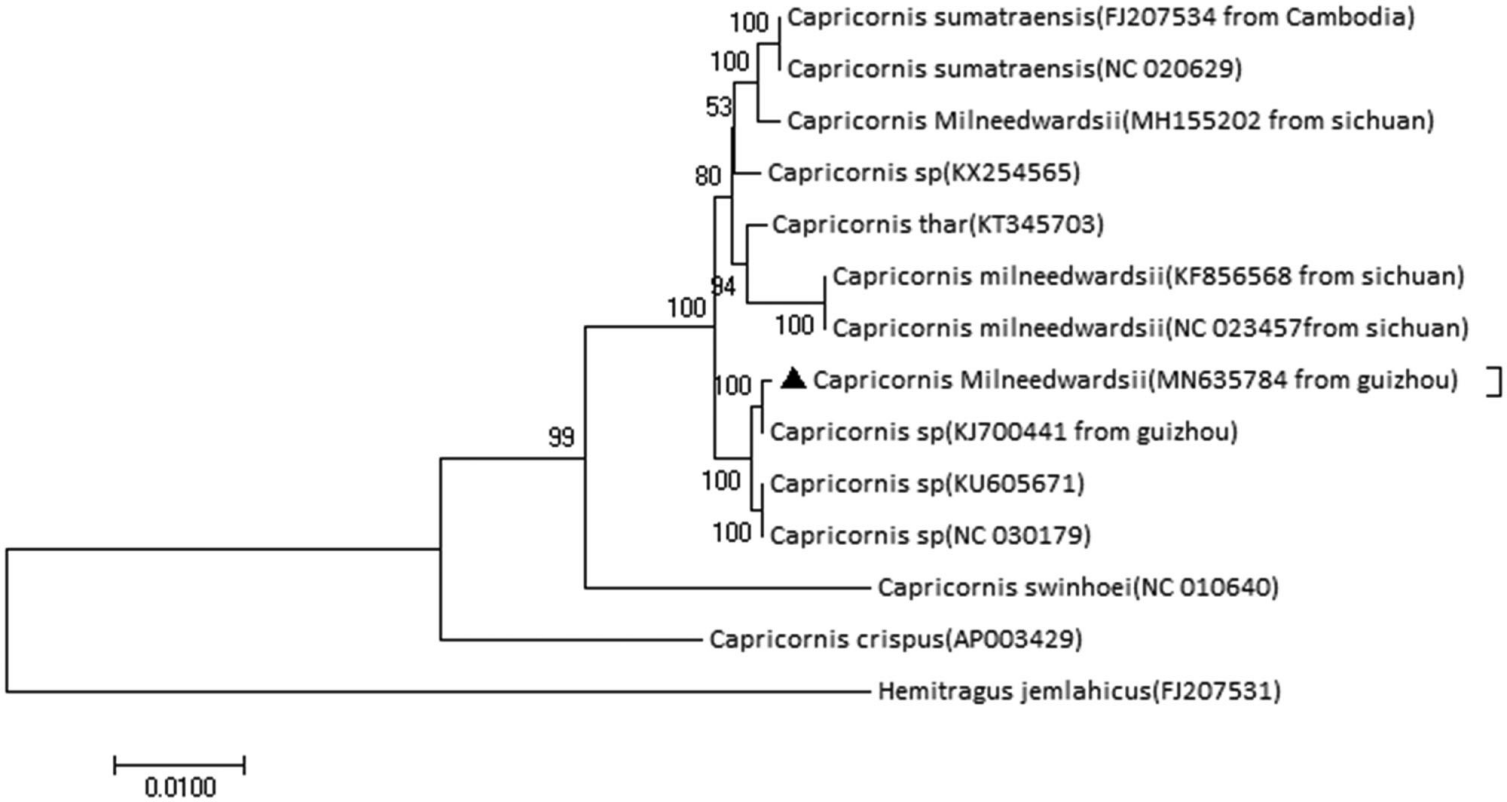
Maximum-likelihood (ML) phylogenetic tree based on an alignment comprising of 11 sequences from genus *Capricornis* and *Hemitragus jemlahicus* as an outgroup. The triangle indicates the sequence from this study. Other sequences were downloaded from NCBI with GenBank accession numbers in the brackets.

## References

[CIT0001] Dou H, Zhang L, Li C, Mu J, Wang T, Ge J, Feng L. 2016 The complete mitochondrial genome of *Capricornis* sp., possible a new species of Serow from Guizhou, China. Mitochondrial DNA Part A. 27(2):848–849. 10.3109/19401736.2014.919471 24865899

[CIT0006] Duckworth JW, Steinmetz R, Pattanavibool A. 2008. *Capricornis milneedwardsii*. The IUCN Red List of Threatened Species. 2008:e.T3814A10101852.

[CIT0003] Hassanin A, Ropiquet A, Couloux A, Cruaud C. 2009. Evolution of the mitochondrial genome in mammals living at high altitude: new insights from a study of the tribe Caprini (Bovidae, Antilopinae). J Mol Evol. 68(4):293–310. 19294454 10.1007/s00239-009-9208-7

[CIT0004] Kumar S, Stecher G, Li M, Knyaz C, Tamura K. 2018. MEGA X: Molecular Evolutionary Genetics Analysis across computing platforms. Mol Biol Evol. 35(6):1547–1549. 29722887 10.1093/molbev/msy096 PMC5967553

[CIT0005] Smith AT, Xie Y. 2009. A Guide to the Mammals of China. Changsha (Hunan): Hunan Education Press; p. 493–495.

[CIT0007] Wang S, Xie Y. 2008. China species red list. Beijing (China): Higher Education Press.

